# Optimization of 3D Extrusion-Printed Particle-Containing Hydrogels for Osteogenic Differentiation

**DOI:** 10.1021/acsomega.4c10515

**Published:** 2025-04-10

**Authors:** Stephanie E. Doyle, Deirdre Winrow, Fiona Buckley, Elin Pernevik, Martin Johnson, Kerry Thompson, Linda Howard, Cynthia M. Coleman

**Affiliations:** † College of Medicine, Nursing and Health Science, School of Medicine, Regenerative Medicine Institute (REMEDI), University of Galway, County Galway, Galway H91 W2TY, Ireland; ‡ CELLINK Bioprinting AB, Långfilsgatan 7, Gothenburg 412 76, Sweden; § Zoan Nuáil Teoranta T/A Zoan BioMed, The Hatchery Building, Cloonacarton, Recess, Galway H91 VW58, Ireland; ∥ College of Medicine, Nursing and Health Science, School of Medicine, Anatomy Imaging and Microscopy, University of Galway, Galway H91 W5P7, Ireland

## Abstract

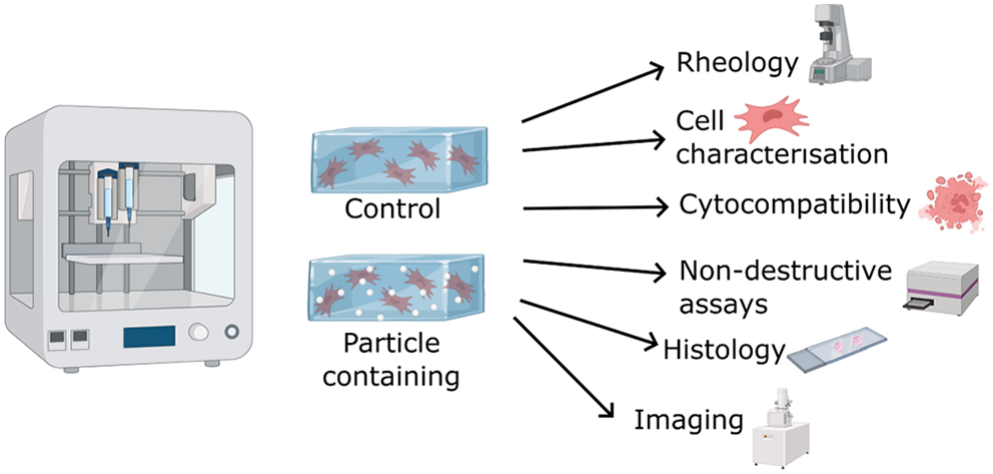

There is a continued increase in demand for novel bone grafting substitutes to reduce reliance on and address challenges associated with allograft and autograft bone grafts. Current synthetic bone grafting substitutes exhibit low mechanical strength and bioactivity, which has inspired the development of novel grafting materials. Accelerating the translation of new bone graft substitutes requires workflows for high-throughput fabrication and analysis of particle-containing models. This study utilized 3D sacrificial printing for the fabrication of reproducible, cellular scaffolds containing tricalcium phosphate (TCP), hydroxyapatite (HA), or natural coral particles. High-throughput analysis of the cellular scaffolds included quantifying cell metabolism, viability, and calcium consumption, as well as nondestructive analysis of collagen accumulation and destructive methods for assessing cell number and morphological changes. Both particle- and non-particle-containing inks sustained cell metabolism with low and decreasing cell death for 7 days post-printing. Collagen staining, scanning electron microscopy imaging, and calcium and collagen quantification suggested that, under osteogenic induction conditions, cells migrated to the surface of the scaffolds and formed a sheet of cells and a collagen-containing extracellular matrix, thereby indicating osteogenic differentiation. The workflow described herein enables the creation of *in vitro* models to study the osteogenic nature of new bone grafting substitute materials. High-throughput printing combined with non-destructive screening techniques resulted in reduced time, resources, and associated costs and could be applicable to a broader range of cell types.

## 1 Introduction

In 2021, the bone graft and bone substitute market was worth 2.72 billion euros globally.^1^ As allograft bone has a limited supply, the demand for alternative substitutes continues to grow. With the increased demand for substitutes comes the need to translate new bone graft substitutes into the clinic, which is traditionally a slow, laborious, and expensive process.

Existing 3D models of osteogenesis include *in vivo*, *ex vivo*, scaffold-containing, or scaffold-free *in vitro* models. Even the most basic *in vivo* model for osteogenesis, subcutaneous implantation in a murine model, is long in duration, requires significant dedication of time by the research team, and is expensive.^2,3^*Ex vivo* models utilizing human or animal tissue can offer a way to study the interaction of the therapy with the native tissue if the appropriate tissue can be sourced. While not achieving the same levels of complexity as the *in vivo* or *ex vivo* models, *in vitro* models offer the advantages of time and cost savings, the capacity to increase sample numbers with relative ease, and the ability to collect results during the culture period.

*In vitro* models of osteogenesis offer the opportunity to build sophisticated three-dimensional (3D) systems to evaluate how a biomaterial would respond *in vivo*. These models can include cells, a physical network for cells to be seeded onto or embedded within, and cell differentiation factors, such as growth factors or ceramic particles. While various cell types may be appropriate in an *in vitro* model, when considering the clinical translation of bone graft substitutes, a human cell source is the most relevant.^4−7^ There are advantages and disadvantages to using human primary or immortalized cells. Primary cells offer great potential for personalized models and high clinical relevance, while immortalized cells have greater longevity with inherently less variability and are thereby more reproducible.^8,9^ The cells can be seeded onto a rigid scaffold, embedded within a softer bioink, or a combination of the two. A stiff scaffold delivers additional mechanical support but can limit proliferation and the ability of cells to reorganize the environment, while a hydrogel provides an environment for greater cell spreading and proliferation.^10,11^ Either option enables the incorporation of differentiation factors. Differentiation factors can be grouped into short- or long-lasting categories. Short-lasting factors include growth factors, hormones, vitamins, etc., with free growth factors commonly having a half-life in the range of minutes to hours.^12−14^ This can be increased when encapsulated or functionalized.^12,14^ Alternatively, particles such as HA, TCP, demineralized and/or decellularized bone, bioglass, or coral have longer stability. For example, TCP was shown to have less than 1% weight loss after 28 days in phosphate-buffered saline (PBS) *in vitro*.^15−17^

TCP and HA are widely used in research and therapeutics for their cytocompatibility, osteoconductivity, and biomimicry to native bone.^18−20^ Coral is a lesser-explored material but is rich in calcium, has mechanical properties in the range of trabecular bone, and allows for cell adhesion while also being osteoconductive and cytocompatible.^21^ A large particle size of 1–2 mm coral has been shown to support more rapid osteogenesis and vascularization *in vivo* compared to synthetic biomaterials.^21^

Therefore, while ceramic-based particles are a suitable bone graft substitute, they can introduce technical challenges in the analysis of *in vitro* models. Calcium-containing ceramics make tracking osteogenic differentiation via calcium staining or quantification challenging.^22,23^ Additionally, internal imaging of 3D bioinks already presents difficulties, which increase when adding particles due to the opacity of the structure or the contribution to the inherent autofluorescence within the sample.^22^ As a result, multiple models and output measures to assess osteogenesis must be considered.

Challenges in the analysis of 3D particle-containing osteogenic models hinder the development and evaluation of novel bone grafting materials. Destructive assays are those that require manipulation of the sample in such a way that the sample cannot be used for any other purpose. Output analysis based on destructive techniques increases the required number of samples, materials, and study costs. Alternatively, nondestructive assays leave little to no lasting impact on the sample, allowing for continued culture or additional assays to be conducted with the same sample. Destructive techniques still have an important role in a collective set of outcome measures; however, they can be complemented with high-throughput, nondestructive techniques to monitor cell health and differentiation over time.

This study utilized 3D printing to achieve high-throughput fabrication of various cellular bioink structures, including two types of inks with ceramic-based micron particles. A baseline commercial ink, GelXA, either on its own or with TCP, HA, or natural coral particles added, was combined with immortalized human mesenchymal stromal cells. A combination of nondestructive, high-throughput, and destructive analytic methods was used to assess cell number, metabolism, death, calcium consumption, and collagen accumulation. Together, this presents a workflow to fabricate and analyze 3D *in vitro* models for testing new bone graft substitute materials.

## 2 Material and Methods

### 2.1 Materials

GelXA and GelXA Bone were provided to the project by CELLINK (Gothenburg, Sweden). GelXA was composed of gelatin methacryloyl (GelMA), xanthan gum, and alginate. GelXA Bone was composed of GelXA base ink combined with TCP/HA particles. In parallel, GelXA base ink was combined with coral particles, which were provided to the project by Zoan Biomed (Galway, Ireland).^21^

Throughout, GelXA Bone was referred to as “TCP/HA,” and GelXA containing coral was simply referred to as “coral” to emphasize the active, variable component of the bioink when compared to the baseline GelXA control. Two sizes of coral particles were used: small coral, with 90% of particles under 37.5 μm, and large coral, with 90% of particles under 117 μm. All inks contained lithium phenyl-2,4,6-trimethylbenzoylphosphinate (LAP), which facilitated photo-cross-linking at 405 nm. The particles were dispersed into a sterile 0.5% LAP solution (in PBS) using a 200 μm mesh as a sieve, while swirling the mix bucket, and speed-mixed at 2000 rpm for 3 min. Finally, warm GelMA (50% degree of methacrylation) and alginate/xanthan gum solutions were added to the warm particle dispersion, which was speed-mixed for 1 min at 1500 rpm and transferred to syringes for further use. (Concentrations are proprietary information from CELLINK Bioprinting AB.) Pluronic-127 was sourced as a sterile 40% w/v solution in water from CELLINK.

### 2.2 Mesenchymal Stromal Cell Culture Expansion

The immortalized human bone marrow mesenchymal stromal cells (iMSC3; Applied Biological Materials, Richmond, Canada) were cultured in growth medium containing α-minimal essential medium with GlutaMAX (Gibco, Waltham, USA), supplemented with 10% fetal bovine serum (FBS; Sigma-Aldrich, Burlington, USA) and 100 U/mL penicillin and 100 μg/mL streptomycin (Gibco) at 37 °C with 5% CO_2_. iMSC3 cells were passaged before reaching approximately 85% confluency. Cell doubling rates were calculated based on a logarithmic scale of the fold increase between cells plated and cells harvested.^24^

### 2.3 3D Printing

Scaffolds were printed using a CELLINK BIO X printer with pneumatic print heads. Acellular inks were printed directly as supplied. Cellular inks were combined with iMSC3 cells at 5–10 million cells per milliliter, depending on the experiment, at a ratio of approximately 1:20 cell volume to ink volume. All scaffolds were printed via sacrificial printing. First, a rectangular 5 × 5 × 1 mm^3^ sacrificial square border was printed using Pluronic-127 at 37 °C, 120 kPa, 4 mm/s, and a 45 °C print bed temperature, into a 24-well adherent-treated plate (Sarstedt, Nümbrecht, Germany). The print bed temperature was then reduced to 15 °C before the acellular or cellular ink was extruded into the center of the sacrificial border for 8 s. There was a 5-s pause to allow the ink to fill out the entire area before photo-cross-linking for 30 s with 405 nm visible light at an irradiance of 43 mW/cm^2^. GelXA, TCP/HA, and small granule coral inks were printed with a 23G needle (CELLINK), while Pluronic-127 and large granule coral were printed with a 22G needle (CELLINK). To dissolve the sacrificial border, cold PBS was washed over the cross-linked structure and brought to room temperature. This process was repeated 3 times in total to ensure the complete removal of the Pluronic-127. Scaffolds were detached from the 24-well plate and transferred to a 48-well plate (Sarstedt) before cell culture media was added. Scaffolds specifically fabricated to be weighed were manually removed from the Pluronic-127 border with forceps and immediately placed on a balance (AS 220.R2, RADWAG, Radom, Poland) before coming into contact with any liquid and swelling. Scaffolds were then submerged in PBS overnight before weighing again to obtain the post-swelling weight (*n* = 4).

### 2.4 Rheology

The rheological characterizations were performed using an HR-10 rheometer (TA Instruments, New Castle, USA), equipped with an upper 20 mm Peltier-plate geometry for temperature control and a bottom borosilicate glass plate enabling *in situ* photo-cross-linking, separated by a 500 μm gap. The shear-dependent viscosity was measured through rotational steady-state flow sweeps at 30 °C from 0.01 to 500/s shear rates, simulating conditions at rest and during extrusion. The temperature dependence was assessed using oscillation temperature ramps from 30 to 21 °C at a rate of 1 °C/min, 0.5% strain and a frequency of 0.5 Hz. After reaching 21 °C (simulating the cooling of constructs post-printing), scaffolds were photo-cross-linked *in situ* using a 405 nm LED module at 43 mW/cm^2^ for 15 s, while storage modulus data was continuously collected. All measurements were conducted on two separate bioink batches; flow sweeps and temperature ramps were performed in triplicate, and photo-cross-linking measurements were conducted with *n* = 4 from two batches.

### 2.5 Particle Size Analysis

The size distribution of TCP, HA, or coral particles was assessed using a Mastersizer 3000 (Malvern Panalytical Ltd., Malvern, England) with the hydro medium volume unit. The protocol was run as per the manufacturer’s instructions with the following key parameters: nonspherical particle type, material (for refractive index and density) specified as TCP with a refractive index of 1.63 and density of 1 g/cm^3^, dispersant of distilled water, red and blue measurements, 10 s for both background and sample, 5 measurements, 5-s delay between each measurement, obscuration at 10% lower limit 20% upper limit, and speed set at 2000 rpm with the general purpose analysis model. Dry particles were added directly to the distilled water in the unit until the obscuration value of 10–15% was reached, after which the program was started. All measurements were performed with a total of *n* = 10 measurements over two discrete experiments.

### 2.6 Scanning Electron Microscopy (SEM)

#### 2.6.1 Preparation of particles

Samples for analysis were dry upon receipt, and fixation was not required. Particles from the representative groups were distributed onto double-sided carbon tabs on aluminum stubs and sputter-coated with 10 nm of gold for 120 s at a current of 25 mA using a Quorum Q150R ES Plus Sputter Coater (Quorum Technologies, Laughton, England). Secondary electron images were collected using a Hitachi S-4700 (Hitachi, Tokyo, Japan) under high vacuum with an accelerating voltage of 5 kV. All processing and subsequent imaging were performed in the Anatomy Imaging and Microscopy Facility, School of Medicine, University of Galway.

#### 2.6.2 Fixation of Acellular Scaffolds and Bioinks

Scaffolds were placed into electron microscopy fixative (2% glutaraldehyde (SERVA Electrophoresis GmbH, Heidelberg, Germany) + 2% paraformaldehyde (MP Biomedicals, Santa Ana, USA) in 0.1 M sodium cacodylate buffer, pH 7.2 (Sigma-Aldrich)) overnight at room temperature. The fixative was removed, and the scaffolds were rinsed in 0.1 M sodium cacodylate buffer and then stored in fresh 0.1 M sodium cacodylate buffer until further processing, as described below.

#### 2.6.3 Freeze-fracture

Scaffolds were freeze-fractured to achieve a more accurate representation of their internal structure. Samples were incubated overnight in a cryoprotective solution of 2.3 M sucrose (Sigma-Aldrich) before being placed in open cryogenic tubes containing 30% ethanol (Honeywell, Charlotte, USA). The samples were then lowered into liquid nitrogen in a cryobox via a cryo-transfer unit using insulated forceps to snap-freeze. Some scaffolds fractured on contact with the liquid nitrogen, while the remaining ones were manually fractured with forceps. Post-fracture, the samples were dehydrated through a series of graded ethanol steps (10 min per step), and upon completion, critical point drying was carried out.

#### 2.6.4 Critical Point Drying

Freeze-fracture fragments were then placed in mesh baskets or plastic dehydration pots. The samples were subsequently placed into the critical point drying sample chamber and submerged in ethanol, which was gradually replaced with liquid CO_2_, in a series of 18 cycles for a total of 2.5 h until the critical point of the liquid CO_2_ was reached. The dried samples were adhered to stubs using carbon tape, sputter-coated, and imaged as above.

### 2.7 Flow Cytometry

Flow cytometry confirmed cell identity and surface marker expression in accordance with the International Society for Cell and Gene Therapy minimal criteria.^25^ iMSC 3s were washed in fluorescence-activated cell sorting (FACS) buffer (2% heat-inactivated FBS in PBS) and passed through a 30 μm filter (CellTrics, Sysmex, Kobe, Japan). The cells were stained using the BD Stemflow Human MSC Analysis Kit (BD Biosciences, Franklin Lakes, USA) with antibodies (CD90 FITC (Clone: 5E10), CD105 PerCP-Cy5.5 (Clone: 266), CD73 APC (Clone: AD2), CD34 PE (Clone: 581), CD11b PE (Clone: ICRF44), CD19 PE (Clone: HIB19), CD45 PE (Clone: HI30), and HLA-DR PE (Clone: G46-6)) and respective isotype controls, as per manufacturer’s guidelines. The samples were washed twice in FACS buffer and resuspended in 200 μL of FACS buffer for analysis. For dead cell exclusion, a 1:20 dilution of DRAQ7 (Thermo Fisher Scientific, Waltham, USA) was added 1 min before sample analysis. The samples were analyzed on a Northern Lights 3000 spectral flow cytometer (Cytek Biosciences, Fremont, USA) with the SpectroFlo software (Cytek Biosciences), and data analysis was performed using FCS Express (version 7, research edition) (De Novo Software, Pasadena, USA). All measurements were performed in technical triplicate.

### 2.8 Mesenchymal Stromal Cell Differentiation

iMSC3s for two-dimensional (2D) differentiation were seeded into six-well plates at 60,000 cells/well in growth medium and allowed to adhere overnight. After 24 h, the growth medium was removed, and the cells were washed with PBS and replaced with either induction or complete media to initiate osteogenic or adipogenic differentiation. Osteogenic complete media contained low-glucose Dulbecco′s modified Eagle′s medium (Sigma-Aldrich) with 10% FBS and 100 U/mL penicillin, 100 μg/mL streptomycin, while osteogenic induction media additionally contained 100 nM dexamethasone (Sigma-Aldrich), 50 μM ascorbic acid 2-phosphate (Sigma-Aldrich), and 10 mM β-glycerophosphate (Sigma-Aldrich). The same complete and induction media were used for 2D and 3D samples. All media were fully changed twice a week for 22 days for osteogenic induction. Adipogenic complete media contained high-glucose Dulbecco′s modified Eagle′s Medium (Sigma-Aldrich) with 10% FBS, 100 U/mL penicillin, and 100 μg/mL streptomycin, while adipogenic induction media additionally contained 1 μM dexamethasone (Sigma-Aldrich), 100 μg/mL insulin (Roche, Basel, Switzerland), 200 μM indomethacin (Sigma-Aldrich), and 500 μM 3-isobutyl-1-methyl-xanthine (Sigma-Aldrich). After 3 days, adipogenic induction media was removed and replaced with adipogenic maintenance media, consisting of high-glucose Dulbecco′s modified Eagle′s medium, 10% FBS, 100 U/mL penicillin, 100 μg/mL streptomycin, and 10 μg/mL insulin. After 1 day, the maintenance media was again replaced with induction media. This cycle was repeated three times before the iMSC3s were left in the maintenance medium for the final 5–7 days. The total adipogenic culture time was 17–19 days.

### 2.9 Alizarin Red S Staining

The osteogenic potential of the iMSC3s was assessed using Alizarin Red S staining. For 2D cells, the medium was removed, and the cells were washed twice with PBS for 5 min each. The cells were covered with 95% methanol (Sigma-Aldrich) and incubated at −20 °C for 10 min to fix. The plates were washed with distilled water and then covered with 2% Alizarin Red S solution (Sigma-Aldrich) for 5 min. The Alizarin Red S solution was removed, and the cells were washed with distilled water until clear (approximately 5 washes). The stained plates were imaged using an EVOS XL CORE bright-field microscope, model AMEX1000. A single representative image is displayed from three discrete experiments.

### 2.10 Oil Red O Staining

The adipogenic potential of the iMSC-3s was assessed using Oil Red O staining. A working solution of 0.3% Oil Red O in 60% distilled water was prepared and allowed to stand for ten min before being filtered through a Whatman No.1 filter (Cytiva). The media was removed from the cells and washed twice with PBS. The cells were fixed in 10% neutral buffered formalin (Sigma-Aldrich) for 10 min at room temperature, then rinsed with distilled water. The cells were subsequently covered with the Oil Red O working solution for 5 min, after which the Oil Red O was removed, and the cells were rinsed with 60% isopropanol (Sigma-Aldrich). Excess stain was removed by washing the culture with tap water, followed by counterstaining with 10% hematoxylin (Sigma-Aldrich) for one min, then a warm tap water wash before imaging with an EVOS XL CORE brightfield microscope, model AMEX1000. A single representative image is displayed from three discrete experiments.

### 2.11 Necrosis Assay

The culture media in 2D or 3D cultures was changed 24 h before conducting the lactate dehydrogenase (LDH) assay to assess cell death. The manufacturer’s protocol from the LDH assay kit (Abcam, Cambridge, England) was followed. Briefly, lysis buffer was added to samples at a ratio of 1:9 with cell culture media for 45 min. The medium was then collected. An LDH positive control was also prepared following the manufacturer’s protocol. Twenty-five μL of conditioned media or LDH positive control was combined with 25 μL of reaction mix and incubated at room temperature for 30 min in the dark. The absorbance was measured at 490 nm using a Victor X3 plate reader (PerkinElmer, Dublin, Ireland). 2D experiments were performed in technical triplicate, and 3D experiments were performed with a total of *n* = 12 measurements over two discrete experiments.

### 2.12 Cell Metabolism Assay

After removing the conditioned media, PrestoBlue was added to the cell monolayer in a ratio of 1:9 PrestoBlue to media, enabling assessment of cellular metabolism. 2D samples were incubated for 1 h and 3D samples for 4 h at 37 °C, unless otherwise indicated. The supernatant was removed, and 100 μL was transferred to a 96-well plate and read at 550/595 nm excitation/emission using a Victor X3 plate reader. 2D experiments were performed in technical triplicate, and 3D experiments were performed with a total of *n* = 12 measurements over two discrete experiments.

### 2.13 Proliferation Assay

To extract the cells from the 3D cross-linked inks, scaffolds were placed in 2.5% trypsin (Sigma-Aldrich) for 30 min, with mixing after 15 min. To separate the cells from the particles, Ficoll-Paque density gradient media was utilized. The cell-trypsin solution was layered on top of Ficoll-Paque (Cytiva) in a ratio of 3:2 cell solution to Ficoll-Paque. The samples were centrifuged at 400 g for 30 min without braking to minimize phase mixing. The cell-containing solution on top of the Ficoll-Paque, as well as half the Ficoll-Paque, was collected, pelleted by centrifugation at 400 × *g* for 5 min, and resuspended before counting. Cells were counted using Via1-Cassette and NucleoCounter (ChemoMetec, Allerød, Denmark). 2D experiments were performed with a total of *n* = 9 measurements over three discrete experiments, and 3D experiments were performed with a minimum of a technical triplicate.

### 2.14 Calcium Content in Media

The calcium content in the media was assessed using the calcium liquid reagent for diagnostic set (Stanbio, Boerne, USA) following the method of Marchese et al.^26^ The conditioned media was harvested in week 6 of the experiment after 4 days of exposure to the sample. A standard curve of 0–1 μg/mL of calcium was generated as per the manufacturer’s instructions. Ten microliters of control or conditioned medium was combined with 190 μL of working solution for each sample. Plates were immediately read at 595 nm absorbance using a Victor X3 plate reader. Absorbance values were converted to concentration (μg/mL) using the linear equation from the standard curve. Conditioned medium from 3 to 8 wells (median of 6.5 wells) per study cohort was assessed in technical duplicate, creating 6–16 measurements for that bioink formulation.

### 2.15 Soluble Collagen Quantification in Media

The soluble collagen released into the media was quantified using the Sircol-2.0 soluble collagen assay kit (Biocolor, Carrickfergus, Northern Ireland) according to the manufacturer’s instructions, including the optional concentration step before proceeding with the general protocol. The conditioned medium was harvested during week 6 of the experiment following 4 days of exposure to the sample. A total of 300 μL of medium was concentrated using the provided concentration reagent. The samples were incubated overnight at 4 °C and centrifuged at 13,000 × *g* for 20 min, and the supernatant was used for subsequent steps. The sample was divided into technical duplicates, and collagen content was assessed using the manufacturer’s general protocol. A standard curve of 0–200 μg/mL was created. Samples were combined with Sircol dye reagent, incubated with agitation for 30 min at 300 rpm, followed by centrifugation at 1500 × *g* for 90 min. The liquid was removed, and plate wash reagent was added. The samples were centrifuged again at 1500 × *g* for 20 min, and dye release agent was added, followed by incubation with agitation at 700 rpm for 30 min. The plates were read at 555 nm absorbance using a Victor X3 plate reader. Absorbance values were converted to concentration (μg/mL) using the linear equation derived from the standard curve and corrected for the concentration protocol. Conditioned medium from two wells was concentrated and then assessed in technical duplicate, resulting in 8 measurements for each sample.

### 2.16 Cryo-Embedding and Sectioning

Scaffolds were fixed in 1% paraformaldehyde (Sigma-Aldrich) for 4 h at room temperature under gentle shaking and stored in 30% w/v sucrose solution (Sigma-Aldrich) at 4 °C. When ready to be processed, the sucrose solution was replaced with a 1:1 mix of 30% sucrose and O.C.T. Compound (Sakura Finetek Europe, Alphen aan den Rijn, Netherlands) for 30 min at room temperature. Scaffolds were then embedded in the O.C.T. Compound in a cryomold (Agar Scientific, Stansted, England) and flash-frozen in liquid nitrogen. Cryosections of 7 μm thickness were cut using a 6250 Cryostat Microtome (Dakewe, Shenzhen, China) and mounted onto SuperFrost Plus adhesion glass slides (Thermo Fisher Scientific).

### 2.17 Histological Staining

All stains follow the same rehydration protocol: 2 min each in 100%, 95%, 70%, and 50% ethanol.

#### 2.17.1 Von Kossa

After rehydration, the slides were stained with 1% silver nitrate (Sigma-Aldrich) and exposed to ultraviolet light for 8 min, followed by two washes in Milli-Q water for 3 min each before transferring the sections to 2.5% sodium thiosulfate (Sigma-Aldrich) for 5 min. The slides were washed twice in Milli-Q water for 3 min each, counterstained with nuclear fast red (Abcam) for 5 min, and then washed in Milli-Q water for 3 min. The slides were then dehydrated for 2 min each in 70%, 95%, 100% ethanol, cleared in xylene (Lennox, Dublin, Ireland) for two changes of 5 min each, and coverslipped with DPX mounting medium (Sigma-Aldrich). Sections were imaged with a Grundium Ocus slide scanner (Grundium, Tampere, Finland).

#### 2.17.2 Picro Sirius Red

After rehydration, the slides were incubated in distilled water for 2 min, then in Picro Sirius Red solution (Abcam) for 60 min, followed by a quick rinse in 2 changes of acetic acid solution (Abcam) and 1 change of absolute alcohol. The scaffolds were dehydrated with two changes of absolute alcohol for 2 min each, cleared in 2 baths of xylene (Lennox) for 2 min each, and then coverslipped with DPX mounting medium (Sigma-Aldrich). The sections were bright-field imaged using a Grundium Ocus slide scanner (Grundium, Tampere, Finland) or viewed on an Olympus BX43 upright microscope configured for polarized light microscopy. Polarized light images were captured at 10× magnification orthogonally, at 90° to each other, to create images A and B using a fixed exposure and gain. Images A and B were overlaid using the Orientation J Fiji Plugin.

### 2.18 Statistics

Quantitative data are summarized as the mean, with error bars indicating the standard deviation. Statistical significance is determined using GraphPad Prism 8 by first testing for normality using the Shapiro-Wilk test, followed by a one-way ANOVA with multiple comparisons of means and Tukey posttest. Statistically significant changes are marked as * = *p* < 0.05; ** = *p* < 0.01; *** = *p* < 0.001; **** = *p* ≤ 0.0001.

## 3 Results and Discussion

### 3.1 Printing Protocol and Reproducibility

**Figure 1:**
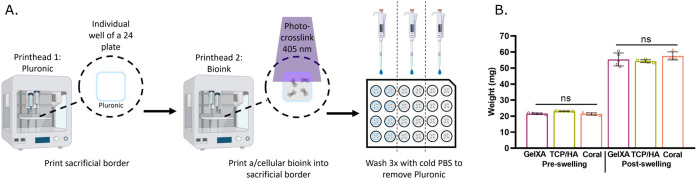
Sacrificial printing technique and reproducibility. (A) Sacrificial printing schematic, figure produced with biorender.com elements, 30/01/2024. (B) Scaffolds produced by sacrificial printing and weighed either immediately after printing or once allowed to swell overnight in PBS, *n* ≥ 3.

All scaffolds were printed using a sacrificial printing technique in which Pluronic was first used to print the sacrificial border before the ink of interest was extruded into the center and photo-cross-linked (Figure 1A). A range of sacrificial materials for biological applications have been used for the creation of outer molds or internal channels, including poly(vinyl alcohol), polycaprolactone, alginate, poly(ethylene oxide), and Pluronics.^27−33^ Pluronic was appealing as a sacrificial material in this workflow because (i) it does not photo-cross-link, (ii) forms a gel at room temperature, and (iii) is a liquid at cold temperatures, allowing it to be removed by washing with cold PBS.^34^ Employing this extrusion method to create 3D constructs resulted in highly consistent scaffold weight, regardless of the inclusion of ceramic particles (TCP, HA, or coral), immediately after printing and after overnight swelling (Figure 1B). The sacrificial printing likely contributed to the reproducibility of construct size and cell number, as the bioink was delivered through one 8-s extrusion of the material rather than the repeated pressure on/off and nozzle movement required for direct extrusion printing. Although successful printing was achieved in this study, strict adherence to print protocols regarding the temperature of the material printed, print bed temperature, and the ratio of cells mixed into the ink was necessary for success. Deviations from these parameters were unsuitable for printing and/or long-term culture.

### 3.2 Characterization of Acellular Inks

The particles contained within the inks were characterized for size, shape, and surface texture, given that these properties can affect the level of influence or interaction with cells.^35−37^ Further assessment of the rheological properties ensured that the addition of the particles was compatible with the properties required for printing acellular or cellular inks.

**Figure 2:**
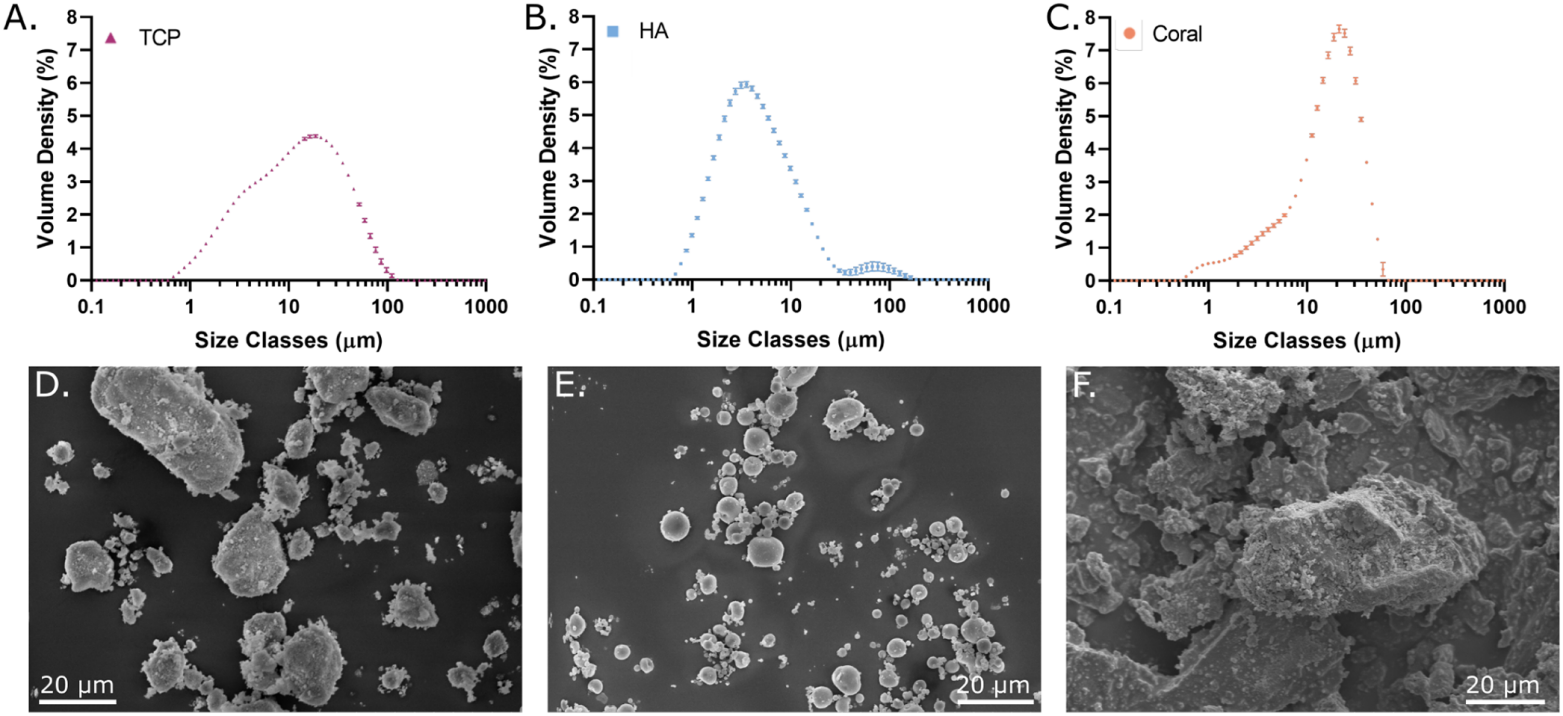
Particle size analysis. (A–C) Particle size analysis for TCP, HA, and coral. Each data point represents the average and standard deviation of *n* = 10 measurements made up from two technical replicates with five measurements each. Data points without visible error bars means the error was too small to show. (D–F) SEM imaging of TCP (D), HA (E), and coral (F) particles.

The particle size distribution of TCP, HA, or coral particles indicated that all particles contained a similar size range, despite differences in their distribution. Analysis found 90% of particles were under 44.8 μm for TCP, 13.7 μm for HA, and 37.5 μm for coral (Figure 2A–C). The SEM imaging of the TCP and coral particles (Figure 2D,F) indicated an irregular, jagged, and potentially rough surface, while HA particles (Figure 2E) displayed a more rounded, smoother surface appearance.

**Figure 3:**
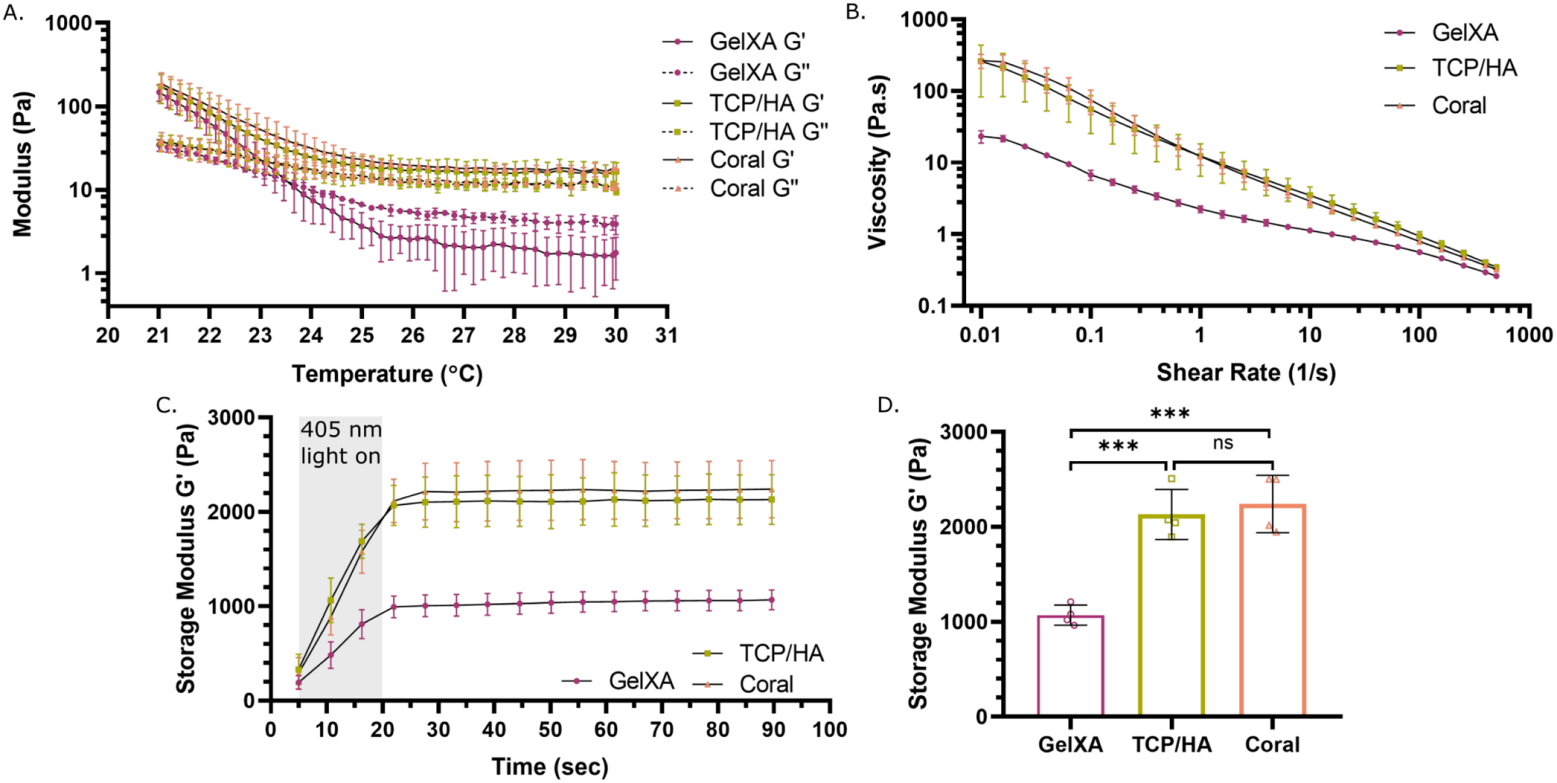
Acellular ink characterization. (A) Oscillation temperature ramps show the temperature dependence of the inks. GelXA displayed a sol–gel transition at 23.5 °C where *G*’ > *G*’’. Above this point the GelMA was melted and contributed very little to the viscosity of the bioink. The presence of particles maintained *G*’ > *G*’’ for the entire range, although above 26 °C the difference was small. At 21 °C the bioinks were thermally gelled. (B) Steady state flow sweeps at 30 °C from 0.01 to 500/s shear rates showed the shear thinning behavior of the bioinks required to minimize shear stress on cells during printing. Presence of particles increased the viscosity compared to pure GelXA. (C) Oscillation time sweep where 15 s of 405 nm light was applied after 5 s (gray box), after reaching 21 °C. (D) Final storage modulus, where all bioinks show at least a 5-fold increase in stiffness after photo-cross-linking. For (A) and (B) *n* = 3, and (C) and (D) *n* = 4 with 2 replicates from 2 independent batches.

In order to characterize the temperature dependence, shear-dependent viscosity, and photo-cross-linking of the inks, rheological analysis was conducted. The temperature ramps in Figure 3A show the temperature dependence of the inks. GelXA displayed a sol–gel transition at 23.5 °C, where the storage modulus, *G*’ became larger than the loss modulus, *G*’’. Above this point, *G*’’, interpreted as the viscous component, was dominant, and GelXA behaved like a liquid. Below this point, *G*’, interpreted as the elastic component, dominated, and GelXA behaved like a gel. This behavior was attributed to the thermoresponsive properties of gelatin. The presence of particles maintained *G*’ > *G*’’ for the entire temperature range. Below approximately 26 °C, *G*’ dominated and the bioinks gelled, whereas above 26 °C, the TCP/HA and coral behaved as viscous liquids.

All bioinks exhibited a decrease in viscosity with increasing shear rates (Figure 3B); this shear-thinning behavior was essential to minimize shear stress on cells during printing and enable extrusion at cell-compatible pressures.^38^ The viscosity data at 30 °C, the printing temperature used in this study, corresponded to the behavior observed during the temperature ramps, as the presence of particles increased the viscosity compared to pure GelXA. This is consistent with other studies where the addition of particles or minerals increases viscosity without compromising the shear-thinning property.^39−41^ For the current application, where the bioinks were not required to print defined filaments but rather to create constructs filled within a defined space established by the sacrificial border, the printing temperature was chosen well above their thermal gelling temperature. However, for filament extrusion, a higher viscosity with an extrusion temperature just above the gel point (where *G*’ starts to increase) would be necessary for print fidelity.

Finally, to ensure the long-term stability of the printed structures, photo-cross-linking was performed to covalently bond the GelMA polymer. This was achieved by exposing the bioinks containing the LAP photoinitiator to 405 nm light, initiating a polymerization reaction. This cross-linking led to an irreversible increase in the storage modulus over 15 s of exposure, as observed in Figure 3C. The photo-cross-linking was functional both with and without the presence of particles, eliminating concerns about optical shielding by the white opaque particles. The inclusion of particles again increased the storage modulus during and after photo-cross-linking, with a final *G*’ of 2.13 ± 0.26 kPa for TCP/HA and 2.24 ± 0.30 kPa for coral, compared to 1.07 ± 0.11 kPa for pure GelXA (Figure 3D), which is consistent with the literature when various particles are added to hydrogels.^42,43^

### 3.3 iMSC3 Characterization

**Figure 4:**
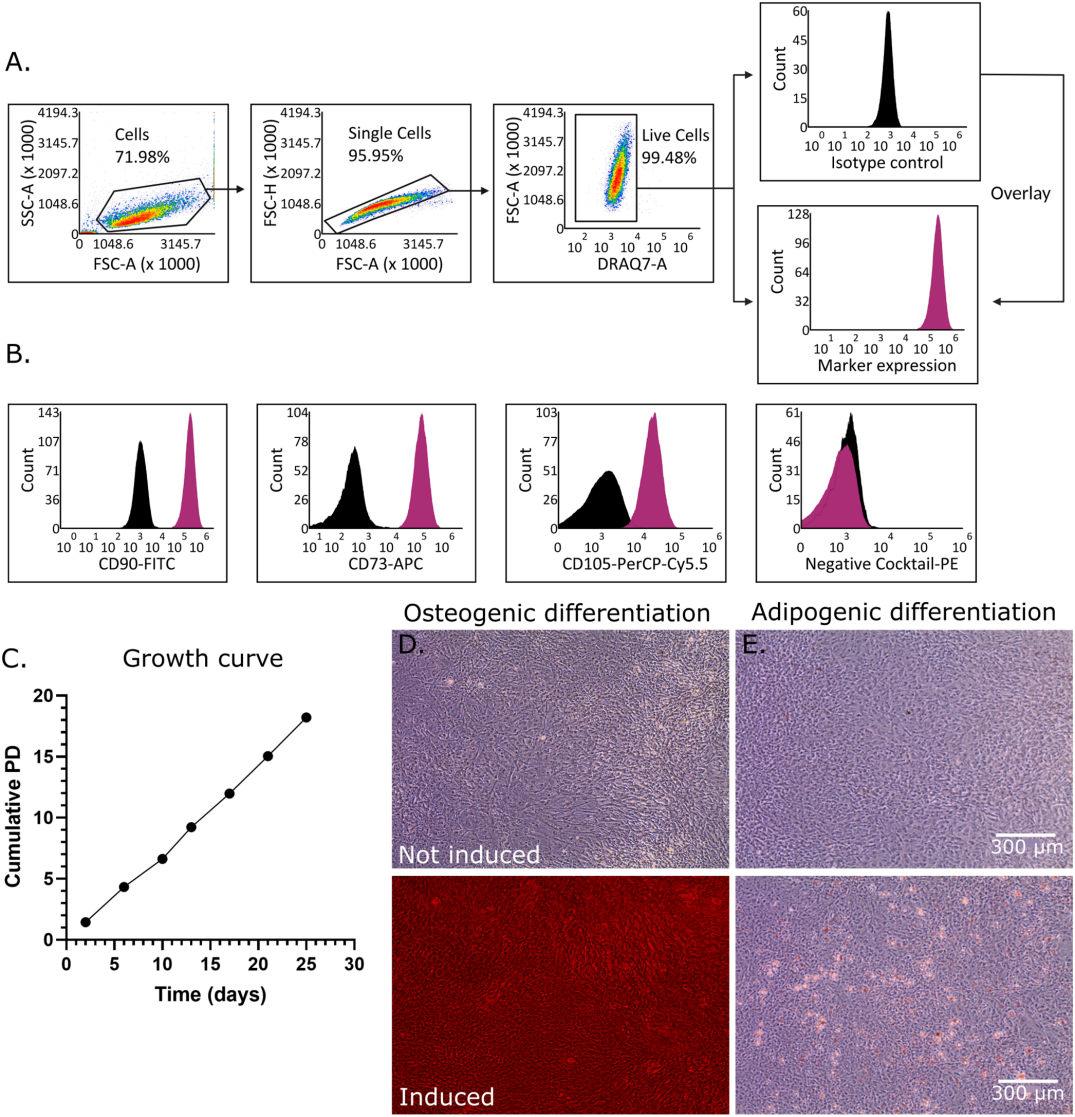
iMSC3 characterization. (A,B) Surface marker characterization of iMSC3s using the ISCT panel of markers. (A) Representative gating strategy for flow cytometric analysis of iMSC3 surface marker expression. (B) Histograms showing the surface marker profile of the iMSC3s. Black histograms represent each isotype control, while purple overlays represent stained iMSC3s. The negative cocktail contains CD34-PE, CD11b-PE, CD19-PE, CD45-PE, and HLA-DR-PE. (C) Cells were cultured in growth medium with the cumulative population doublings (PD) calculated based on a logarithmic scale of the fold increase between cells plated and cells harvested, *n* = 1. (D,E) Assessment of dual lineage potential with osteogenic (D) and adipogenic (E) differentiation potential. Osteogenic differentiation was assessed by staining with Alizarin Red S after 22 days and adipogenic with Oil Red O after 19 days; scale bar in (E) applicable to (D).

The iMSC3s were confirmed to be multipotent progenitor cells by characterizing their cell surface phenotype, growth profile, and multilineage differentiation potential. To assess the surface marker expression of iMSC3s by flow cytometry, cells were gated first by discrimination from debris, followed by exclusion of doublets and dead cells (Figure 4A). The flow cytometry results confirmed the surface marker expression of CD90, CD73, and CD105, and the lack of surface marker expression of CD34, CD11b, CD19, CD45, and HLA-DR by iMSC3s, consistent with the minimal criteria for defining multipotent mesenchymal stromal cells established by the International Society for Cellular Therapy (Figure 4B).^25^

As an immortalized cell line, the iMSC3s should retain, at a minimum, a long-term proliferation capability;^44,45^ therefore, the growth kinetics of in-house iMSC3s were profiled. Exponential proliferation was maintained over 25 days of culture (Figure 4C) where primary bone marrow-derived MSCs can begin to plateau as early as 10 days in culture,^24,46,47^

A characteristic of primary mesenchymal stromal cells is their multilineage potential. The iMSC3s showed both osteogenic (Figure 4D) and adipogenic (Figure 4E) differentiation potential; however, our data showed a more robust response to osteogenic stimulation than adipogenic stimulation.^48^ These results are consistent with the original description of the cell line and subsequent published work demonstrating that adipogenic differentiation is possible but can be limited for iMSC3s. However, it can be enhanced, for example, when treated with pharmacological agents for diabetes.^49−51^ Similarly, the osteogenic response can also be enhanced when treated with hydroxycholesterols.^52^

### 3.4 Development and Validation of Cellular Assays for 3D Scaffolds Containing Particles

**Figure 5:**
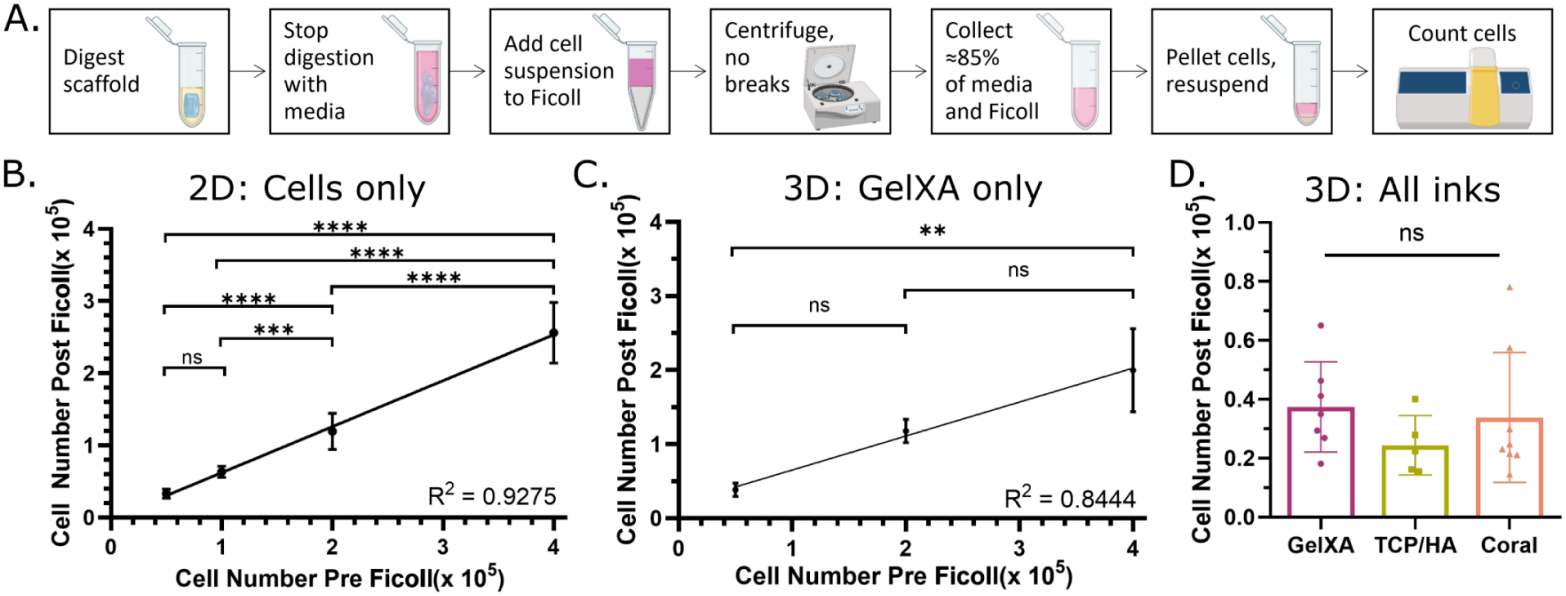
Cell extraction from bioink using Ficoll-Paque. (A) Schematic representation of the protocol. Figure produced with biorender.com elements, 30/01/2024. (B) Control experiment demonstrating even in 2D, cell loss occurs when centrifuging a known number of cells through Ficoll-Paque, *n* = 9. (C) Varying cell number printed in a consistent volume of GelXA before being digested in trypsin, put through Ficoll-Paque, and counted. Large differences in cell number per construct can be detected, *n* = 3. (D) Consistent cell number (≈125,000 cells per scaffold) 3D printed in each of the three bioinks then digested in trypsin, put through Ficoll-Paque and counted, demonstrating a consistent cell yield regardless of the bioink, *n* ≥ 5.

Transitioning from 2D cell characterization to 3D scaffolds requires the development, optimization, and validation of assays, including assessing cell number, metabolism, and death. Assessing cell number in a hydrogel scaffold is challenging due to the inclusion of particles, given their often-similar size and shape to cells and their potential to increase the opacity of the scaffold. To overcome this, the differences in densities between particles and cells were exploited using Ficoll-Paque. Ficoll-Paque is designed to isolate human mononuclear cells from blood; however, it has also been used in a range of alternative applications.^53−57^ To isolate cells from the bioink, the scaffold was digested using trypsin, and the cell suspension was then layered on top of the Ficoll-Paque and centrifuged (Figure 5A). The particles and bioink precipitated, while the cells remained in the zone between the media and Ficoll-Paque. The cells were then collected and counted without interference from the particles; however, there was a high level of cell loss during the process. Control experiments in 2D were first conducted by adding 50,000 to 400,000 cells to the Ficoll-Paque. The assay was only sensitive enough to statistically determine differences greater than 50,000 cells (Figure 5B). Transitioning to 3D (GelXA bioink) reduced the sensitivity, with cell loss increasing to approximately 70%, thereby requiring a difference of 350,000 cells to statistically detect variations in cell numbers from the scaffold (Figure 5C). However, this finding was independent of the presence or absence of particles in the bioink (Figure 5D). To the best of our knowledge, there is no published literature that digests particle-containing cellular hydrogels and then separates the particles and cells. However, common reagents used to isolate cells from hydrogel inks without particles include collagenase (I, V, VI, P) and trypsin.^58−60^ Published studies by Peerani et al. and Virumbrales-Muño et al. only show the percentage of viable cells, so it is unclear if cells are lost during their isolation.^58,60^ Huang et al. isolated the cells 30 min after fabrication and showed no significant cell loss after using an isolation protocol based on shearing the peptide hydrogel and trypsin.^59^

**Figure 6:**
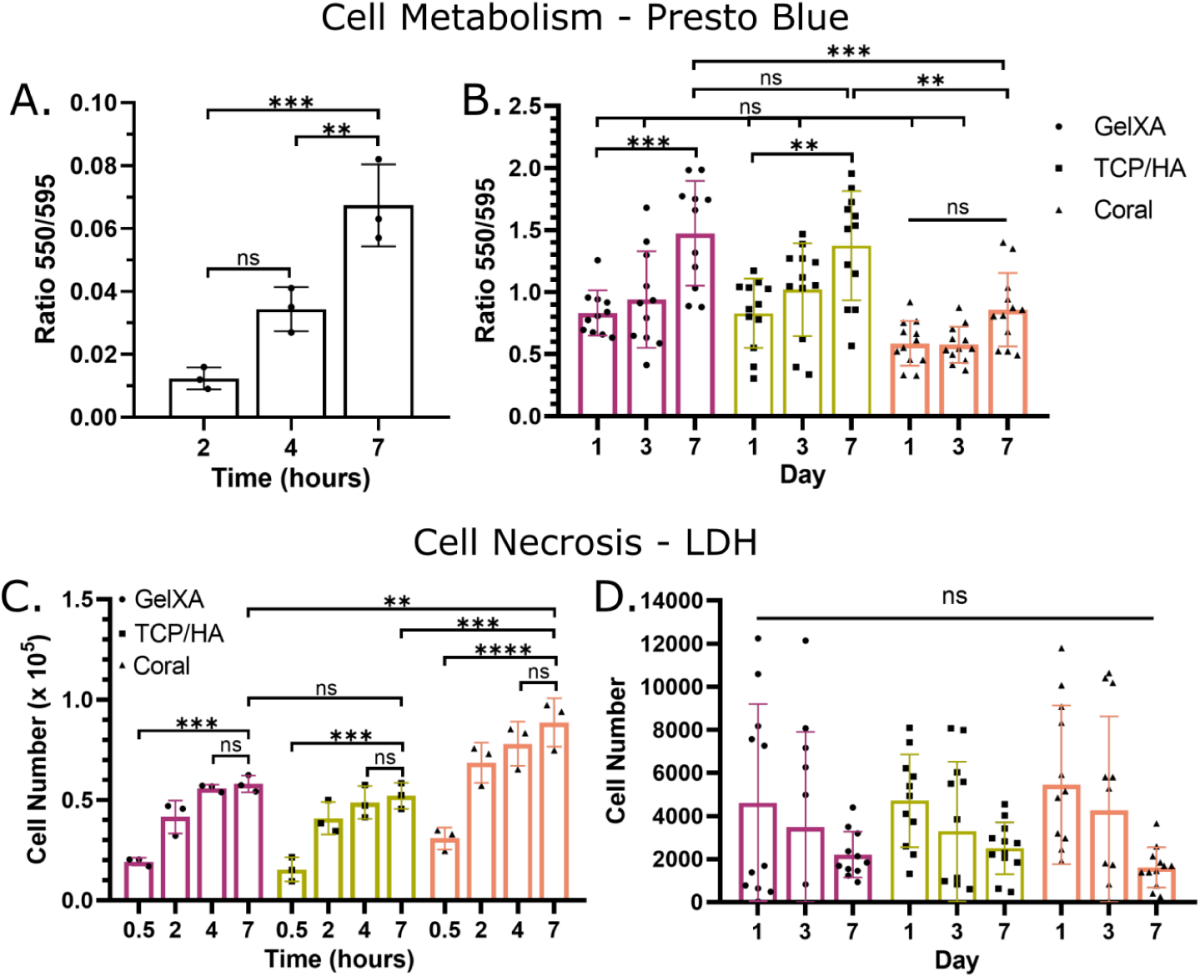
Metabolic and viability assay validation in 2D and 3D. (A-,B) Cell metabolism was assessed using PrestoBlue reagent with all graphs presented as blank corrected values. (A) GelXA cellular scaffolds incubated with PrestoBlue over time to assess the optimal time in 3D, which was identified to be at least 4 h given 2 h was too close to a reading of 0 when the black is subtracted, *n* = 3. (B) Scaffolds incubated for 4 h with PrestoBlue before obtaining an absorbance reading of the supernatant. Scaffolds were assessed 1, 3, and 7 days after 3D printing demonstrating that the printing process did not negatively affect cell metabolism, *n* = 11–12. (C–,D) Cell necrosis was assess using a LDH assay. (C) Cell death was induced in cellular 3D scaffolds with lysis buffer to determine how long in buffer was needed to reach a plateau reading to indicate death of all cells in the scaffold. A plateau was reached after 4 h, *n* = 3. (D) Evaluation of condition media from cell containing scaffolds, each holding approximately 125,000 cells. No significance was found given variability; however, in all conditions, the trend was a decrease in cell death from day 1 to 7, *n* = 11–12.

To ensure the printing process, with or without particles, does not induce cell death, the metabolism and necrosis of the cells in the 3D scaffolds were assessed. First, a standard curve of iMSC3 metabolism in 2D on tissue culture plastic was created, demonstrating low variability and a predictable increase in metabolism with increased cell numbers (Figure S1). Cells in GelXA were then incubated with PrestoBlue for 2–7 h, given signal increases over time, resulting in a stepwise increase in signal detected with time (Figure 6A). Next, all three cellular bioinks were assessed on days 1, 3, and 7 after printing, with no significant difference between any conditions on day 1 and day 3 (Figure 6B). All conditions showed an increase in cell metabolism from day 1 to day 7, although this increase was only significant in the GelXA and TCP/HA conditions. In comparison to the literature, these results demonstrated better stability of cell viability post-printing. Others have shown that primary bone marrow mesenchymal stromal cells (MSCs) in GelXA or a TCP-containing ink experienced a large drop in viability over the first 4–7 days before increasing until day 28.^61^ Meanwhile, primary bone marrow MSCs in an alginate or alginate-GelMA hydrogel showed a viability of ≈70–90% after 7 days post-printing.^62,63^ However, without data presented for day 0/1, it is unclear whether this represents an increase, decrease, or plateau after printing.

Foundation experiments to evaluate cellular necrosis were conducted using the LDH assay first in 2D. The iMSC3s were seeded with 5000–100,000 cells per well, and lysis buffer was then added to kill the cells. A linear increase in LDH signal was recorded with a plate reader, with a statistically significant difference detected starting at 15,000 cells (Figure S1). In transition to analyzing 3D cultures (≈125,000 cells per scaffold), it was first validated that the LDH released by necrotic cells could transfer through the bioink into the conditioned medium, and that the inclusion of particles did not interfere with LDH transfer to the medium. Lysis buffer was added to cellular 3D scaffolds, and aliquots of the medium were sampled over time. There was a stepwise increase in LDH absorbance between 0.5 and 4 h, which plateaued between 4 and 7 h (Figure 6C). The LDH assay can be correlated between 2D and 3D, as Figure 6C confirms the ability of LDH to be released into the culture medium. Therefore, the linear equation in Figure S1 was used to convert absorbance to cell number in Figure 6C,D. In the 7 days after printing, there was no significant difference between any time point or any ink in the number of dead cells recorded in the 24 h prior to the time point (Figure 6D). Despite variability, each ink shows the same trend, with the greatest levels of cell death occurring in the 24 h post-printing and a slight decrease at days 3 and 7. Even the highest individual points, which represent cell death of approximately 12,000 cells, account for less than 10% of the total cell number in each scaffold. Overall, it can be concluded that there are no cytotoxicity concerns with any of the bioink formulations or the printing process. This is consistent with the literature, where the rate of cell death (human primary MSCs, human immortalized MSCs, mouse preosteoblasts) when embedded in hydrogels was maintained or decreased over a period of 7–28 days.^64−67^ Manferdini et al. demonstrated this rate to be approximately 10–15% cell death of human primary MSCs in a xeno-free hydrogel on days 2 and 7.^64^

### 3.5 Osteogenic Differentiation of 3D Scaffolds

To assess the potential for particle-containing bioinks to support iMSC3 osteogenesis, scaffolds were printed and cultured in osteogenic induction or noninduction (control) conditions for 6 weeks. Both calcium and collagen assays were employed to assess osteogenesis, showcasing the ability to identify a suitable assay regardless of the biomaterials used.

Here, a fourth ink was introduced, consisting of the GelXA base ink with larger coral particles. The larger-sized particles were studied for their ability to induce osteogenesis due to size alone or their potential to release calcium, which may differ from the smaller-sized particles .^36,68^ As mentioned, the previous ink, referred to as “coral,” had 90% of particles under 37.5 μm, and from here on, this ink will be referred to as “small coral”. The new ink contains coral particles with 90% of particles under 117 μm (Figure S2) and will be referred to as “large coral”. LDH levels were assessed across the 6 weeks of culture to monitor necrosis levels resulting from printing with a larger particle size (Figure S3). In the 24 h following printing, all conditions had less than 5% cell death, excluding the large coral size, which experienced approximately 13% cell death. This was likely due to challenges in printing, which required the large coral ink to switch from printing with a 23G needle to a 22G needle due to blockages. All remaining time points and conditions displayed equal or lower cell death compared to day 1, with less than 5% cell death from the original seeding density.

**Figure 7:**
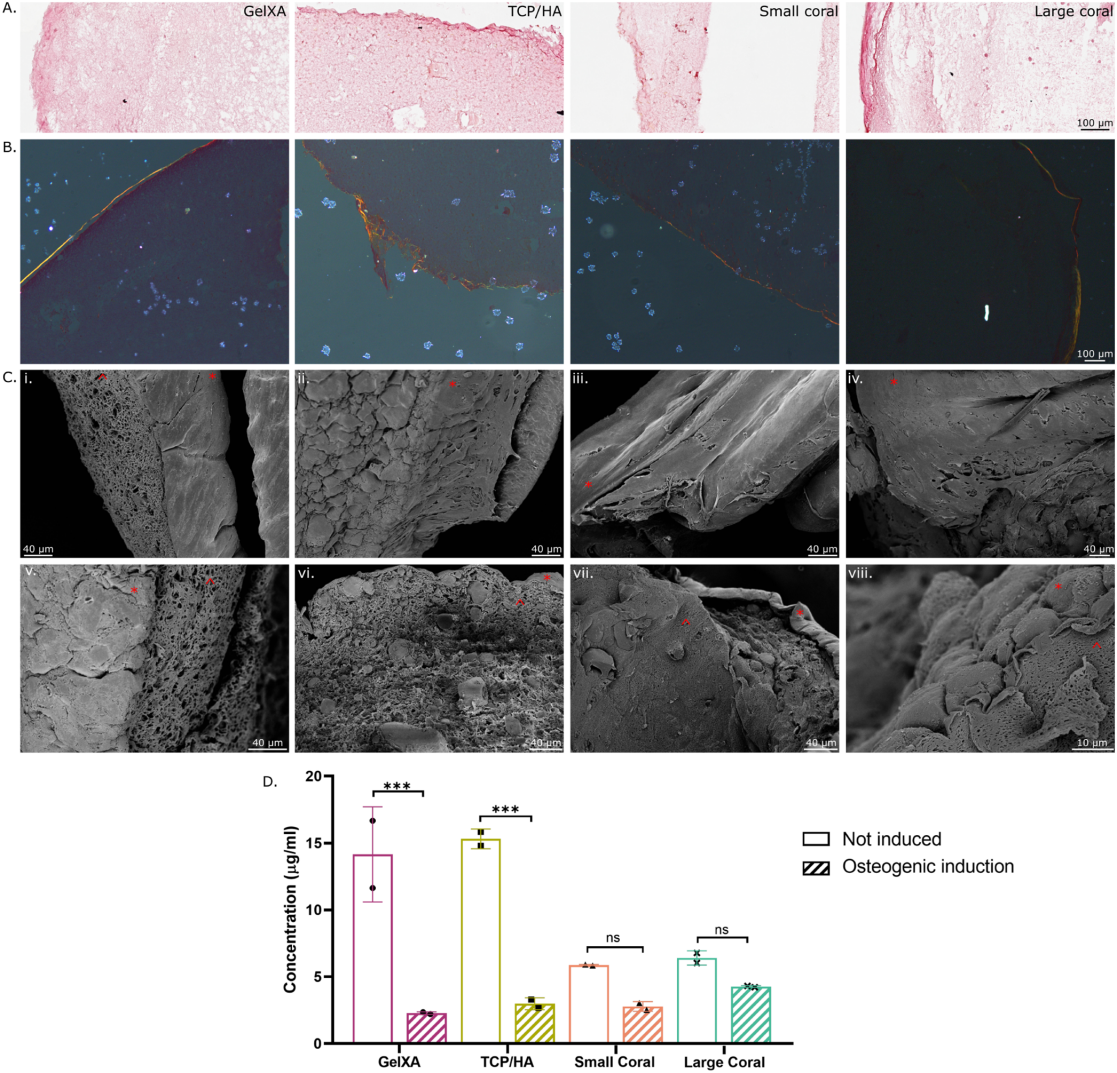
Collagen content of cellular bioinks cultured in osteogenic induction media for 6 weeks. (A) Picro Sirius Red staining imaged under light microscopy. Solid, deep red staining indicated the presence of collagen. (B) Picro Sirius Red staining imaged under polarized light microscopy. Yellow to orange fluorescence indicated collagen type I. (C) SEM imaging. * = outer surface of the scaffold. ^ = internal surfaces exposed by freeze fracturing. (D) Quantification of soluble collagen released into the cell culture media harvested at week 6 and fed 4 days prior, *n* = 2.

Osteogenic differentiation was assessed by collagen imaging, quantification, calcium consumption, and mineralization. Extracellular collagen was visualized using Picro Sirius Red staining (Figure 7A,B) and SEM imaging (Figure 7C) while the soluble collagen released into the culture media was quantified (Figure 7D). The Picro Sirius Red, when imaged under light microscopy, showed consistent background absorbance by the bioinks on day 1 (Figure S4), persisting to week 6 of culture (Figure 7A). Under polarized light, there was no true signal indicating the presence of collagen type I on day 1 (Figure S5). After 6 weeks of culture, the noninduced group displayed a loose ring of positively stained collagen on the edge of the scaffold (Figure S5). In osteogenic conditions, a more pronounced band of staining was observed at the superficial edge of all constructs, especially under the TCP/HA and large coral conditions. Under polarized light (Figure 7B) all conditions contained a combination of yellow and red-stained collagen fibers, indicative of collagen type I. Green staining, indicative of collagen type III, was absent or minimal in all conditions.^69^ Although all conditions had more intense staining on the outer edge of the scaffolds, the bioink containing large coral particles displayed the thickest and most continuous ring.

SEM imaging enabled visualization of the differentiating constructs. In all conditions, a sheet of cells and a ring of extracellular matrix were observed on the outer surface of the scaffolds (indicated by *) (Figure 7C). There was a clear distinction in texture between this dense, smooth outer band and the porous hydrogel (indicated by ^) (Figure 7Cv–viii). A sheet of cells was observable on the outer surface of noninduced scaffolds (Figure S6), however, that band was thicker in differentiated cultures. There was no distinguishable difference in the size or composition of the sheet of cells or matrix between osteogenically differentiated cohorts. The increased migration and/or activity of the cells at the outskirts of the scaffold may be linked to greater accessibility to nutrients from the media or lower scaffold stiffness.^70^

In the TCP/HA scaffolds, the particles were highly pronounced (Figure 7Cvi), while in both the small and large coral conditions (Figure 7Cvii,viii), the particles were less distinguishable. All particle-containing inks had the same weight-per-volume addition of particles, presenting several hypotheses as to why the coral is less visible in the SEM images. All scaffolds were fixed for electron microscopy and then left in a sodium cacodylate buffer for up to 3 months until imaging. All the particle-containing conditions were opaque inks with the same level of opacity maintained after printing and during culture; however, the small coral scaffolds became more transparent by the time of imaging (Figure S7). The pH of the sodium cacodylate buffer is 7.2, similar to cell culture media, while ocean water is approximately 8.1–8.2.^71,72^ Even at a pH reduction to 7.8 from 8.2, the dissolution rate of coral is increased in certain species of coral.^72^ Therefore, the coral particles may have been slowly dissolving, with small particles affected more than the large.

Quantification of soluble collagen released into the cell culture media is a well-validated, nondestructive methodology to assess osteogenesis.^73^ Here, soluble collagen was quantified in control and differentiated cultures after 6 weeks of differentiation (Figure 7D). Overall, the scaffolds in osteogenic induction media released a lower collagen concentration into their culture media compared to the noninduction group. However, this was significant only in the GelXA and TCP/HA groups.

When interpreted with polarized Picro Sirius Red and SEM imaging, it was hypothesized that osteogenic differentiation results in a dense band of collagen around the periphery, which limits the amount of soluble collagen released into the conditioned media. Following this, a low collagen content suggests that osteogenesis is occurring in the osteogenic induction groups. The coral conditions in noninduction media also have a similar collagen content to their counterparts in induction media, which could also indicate osteogenesis or an affinity of the collagen to the coral particles directly.

**Figure 8:**
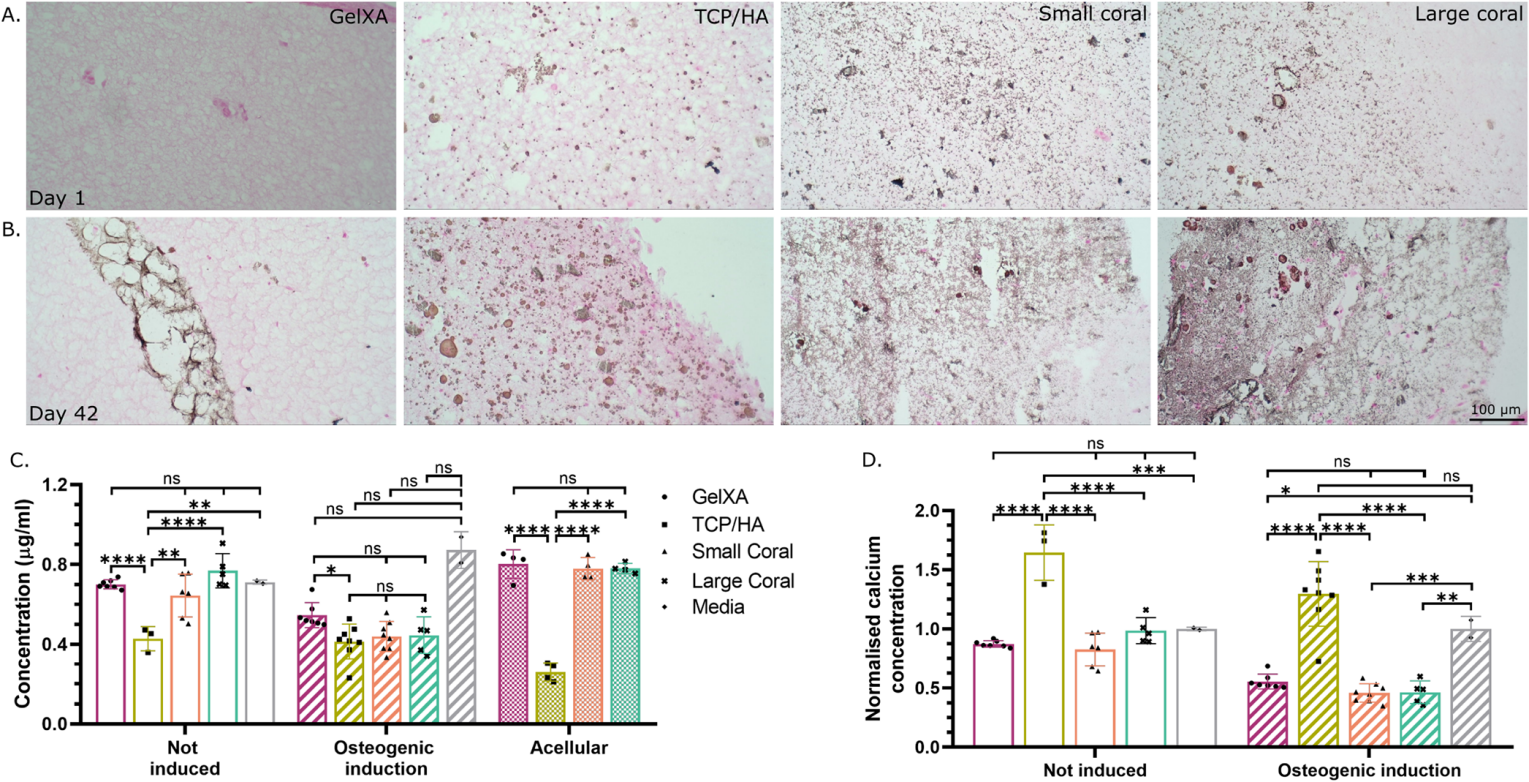
Calcium content of cellular bioinks cultured in osteogenic induction media for 6 weeks. (A,B) Von Kossa staining indicated the presence of calcium with brown deposits. (C–,D) Calcium content of the cell culture media harvested on week 6 and fed 4 days prior. A depletion of calcium from the medium indicates it has been actively sequestered by the cells. (C) The raw data, that is normalized (in D) to media and acellular conditions. *n* ≥ 2.

In osteogenesis, collagen-containing extracellular matrix deposition is followed by calcium-rich mineralization.^74^ Therefore, calcium deposition was assessed through Von Kossa staining (Figure 8A,B) and calcium consumption from the media (Figure 8C,D). The challenge of assessing calcium deposition when working with calcium-containing bioinks is distinguishing between a bioink particle and newly cell-synthesized calcium deposits. The lack of particles in the GelXA scaffolds allowed them to serve as a control. No brown calcium deposits were observed in Von Kossa-stained scaffolds after day 1 (Figure 8A) or week 6 under noninduced conditions (Figure S8). After 6 weeks of osteogenic induction, small and large brown calcium deposits were observed under the GelXA conditions (Figure 8B), demonstrating osteogenic differentiation of the iMSC3s. In the TCP/HA and large coral conditions, an increase in the overall abundance of deposits was observed after 6 weeks of osteogenic differentiation. Only in TCP/HA was this increase seen in both induction (Figure 8B) and noninduction (Figure S8) media conditions which can therefore be attributed to the TCP/HA particles. In both coral conditions (week 6, induced), a brown “dust” covering was present, with only some individual particles easily distinguishable. However, as the coral particles were shown to be irregularly shaped (Figure 2F), it is hypothesized that the spherical deposits that increased in concentration over time were synthesized by differentiating cells.

Depletion of calcium from the conditioned medium was used herein as a quantitative indicator of osteogenic differentiation. This methodology, developed by Marchese et al., is nondestructive and high-throughput compatible.^26^ When assessing background control calcium absorption from the media by the acellular bioinks, the GelXA, small and large coral exhibited a similar pattern of calcium depletion, comparable to basal media, while the TCP/HA bioink showed significant absorption of calcium from the medium. It appears that the presence of the TCP/HA particles themselves is responsible for absorbing calcium from the medium and incorporating it into the scaffold. A study by Šupová et al. demonstrated the ability of HA particles in media to reduce the free calcium ion concentration to zero within a day, while TCP particles reduced the free medium calcium concentration by approximately 50%.^75^ Similarly, Petrakova et al. demonstrated the ability of octacalcium phosphate to precipitate an additional phase of calcium mineral onto the surface of existing particles following exposure to media.^76^ Under cellular conditions, the noninduced GelXA and the coral-containing bioinks exhibited a similar calcium concentration to basal media that was not exposed to cells (Figure 8C). However, the TCP/HA bioink demonstrated a significant drop in calcium in the conditioned medium, which followed the same trend observed in the acellular condition (Figure 8C). In the osteogenically induced scaffolds, all formulations experienced a drop in calcium concentration compared to the basal media that was not exposed to cells.

To account for calcium depletion by the TCP/HA material and compare across conditions, calcium depletion by cellular scaffolds was normalized to the media and acellular constructs made from the same bioink (Figure 8D). After normalization, the TCP/HA in both the osteogenic induction and noninduction media has the same calcium concentration, which is greater than the basal media that was not exposed to cells. Therefore, there may be more complex interactions occurring within the TCP/HA material that cannot be accounted for with this type of normalization. Direct comparison of each bioink with the media showed a statistically significant depletion of calcium from the medium only in those in induction media (excluding TCP/HA), indicating osteogenesis (Figure 8D). Previous *in vivo* findings indicated that the culture of MSCs with coral particles led to earlier bone formation compared to TCP/HA, which could be consistent with the findings presented here.^21^

In comparison to the literature, to the best of our knowledge, there is one other published study that examines GelXA TCP/HA using assays for osteogenic differentiation.^61^ After 3 weeks in culture *in vitro*, there was a significant increase in bone volume compared to the acellular material, as assessed by microcomputed tomography. The results suggest that GelXA TCP/HA can support osteogenic differentiation; however, it was inferior to the other inks used in the study.^61^

It is worth noting that alkaline phosphatase (ALP) secretion is a standard marker for MSC osteogenic differentiation. However, it was not used in this study because the release of ALP into the medium by 2D osteogenically differentiating iMSC3s was not observed, even when control primary MSCs did release detectable quantities of ALP into the medium (Figure S9). Mussa et al. reported similar findings, where ALP gene and protein expression were suppressed in iMSC3s as compared to primary cell types.^77^ Similarly, Marchese et al. compared ALP activity in primary MSCs and iMSC3s under osteogenic differentiation and found that the induced primary MSCs had a 5-fold increase compared to the noninduced group after 7 days, while the iMSC3s had less than a 2-fold increase in ALP expression after inducing differentiation.^26^ Therefore, while ALP activity in the media has the potential to be a great high-throughput, nondestructive way to track osteogenic differentiation, it may not be suitable for all cell types, including immortalized cells.

## 4 Conclusion

This study demonstrated the ability to 3D print various particle-containing cellular bioink scaffolds in a high-throughput manner. These scaffolds were able to support cell metabolism and exhibited low cell death over both 6 and 7 weeks. A method for extracting the cells from the scaffolds and separating them from the particles was developed using density-separating media. This approach allowed for cell counting and the potential to assess cells in further downstream applications (e.g., flow cytometry). When cultured for 6 weeks in osteogenic induction media, it was observed that the cells formed a sheet on the outer surface of the scaffolds and a band of extracellular matrix, including collagen. An increase in mineralized calcium deposits was observed after culture in induction media, and a significant change in calcium depletion from the media occurred in the GelXA and coral induction groups, indicating osteogenesis. The lack of ALP production in the immortalized human MSCs underscores the importance of developing and using alternative assays, such as those measuring collagen and calcium. These assays, along with ALP (where suitable), can be used independently or in combination and represent a suite of high-throughput, nondestructive methods to assess osteogenesis. This approach is particularly useful when working with multimaterial dynamic systems and comparing the interactions between particles, media, and secreted proteins. This *in vitro* model offers time and cost advantages by prioritizing nondestructive techniques, such as assessing cell metabolism and necrosis over DNA content or live/dead staining, and secreted collagen over retained collagen in the matrix.^78,79^ While only one cell type is used in the current study, the 3D environment provides the ability to incorporate additional cell types, including osteocytes and osteoclasts, to gain further insights into the interaction of these cells with the grafting material. The model itself lacks vascularization, a key component of bone, but the 3D hydrogel model can be cultured in lab-on-a-chip devices or a chick chorioallantoic membrane assay to investigate this contribution before transitioning to an animal model.^80−82^

Overall, the cells were able to show signs of osteogenic differentiation, but more work is required to determine whether the particles are sufficient to cause this differentiation without stimulus from the media. The workflows developed allow for *in vitro* testing of new bone graft substitute materials.

## Supporting Information Available

2D cell metabolism and necrosis assessment; coral particle size analysis; 3D scaffold necrosis tracking; Picro Sirius Red staining; scanning electron microscopy imaging; particle stability in scaffolds; Von Kossa staining; alkaline phosphatase quantification (PDF)

## Abbreviations

TCP: Tricalcium phosphate
HA: hydroxyapatite
3D: three-dimensional
PBS: phosphate-buffered saline
GelMA: gelatin methacryloyl
LAP: lithium phenyl-2,4,6-trimethylbenzoylphosphinate
iMSC3: immortalized human bone marrow mesenchymal stromal cells
RPM: revolutions per minute
FBS: fetal bovine serum
FACS: fluorescence-activated cell sorting
2D: two-dimensional
LDH: lactate dehydrogenase
SEM: scanning electron microscopy
ALP: alkaline phosphatase
