# Supplementary material for: Optimization of 3D Extrusion-Printed Particle-Containing Hydrogels for Osteogenic Differentiation
Source: ACS Omega. 2025 Apr 10;10(15):15036–51. doi: 10.1021/acsomega.4c10515 (PMC12019730; doi:10.1021/acsomega.4c10515)
Supplement: Supplementary file 1 — ao4c10515_si_001.pdf [file ao4c10515_si_001.pdf]

Optimisation of 3D extrusion printed particle-containing hydrogels for osteogenic differentiation

Stephanie E. Doyle[1], Deirdre Winrow[1], Fiona Buckley[1], Elin Pernevik[2], Martin Johnson[3], Kerry Thompson[4], Linda Howard[1], Cynthia M. Coleman[1]*

1. Regenerative Medicine Institute, School of Medicine, College of Medicine, Nursing and Health Science, University of Galway, Galway City, County Galway, Ireland, H91 FD82

2. CELLINK Bioprinting AB, Långfilsgatan 7, 412 76 Gothenburg, Sweden

3. Zoan Nuáil Teoranta T/A Zoan BioMed, The Hatchery Building, Cloonacarton, Recess, Galway, Ireland

4. College of Medicine, Nursing and Health Science, School of Medicine, Anatomy Imaging and Microscopy, University of Galway, Galway, H91 W5P7, Ireland

***Corresponding Author:**

Stephanie E. Doyle, PhD

Regenerative Medicine Institute (REMEDI) Biomedical Science Building, 1st Floor South

University of Galway, Galway, Ireland H91 W2TY

Email: sdoyle.email@gmail.com

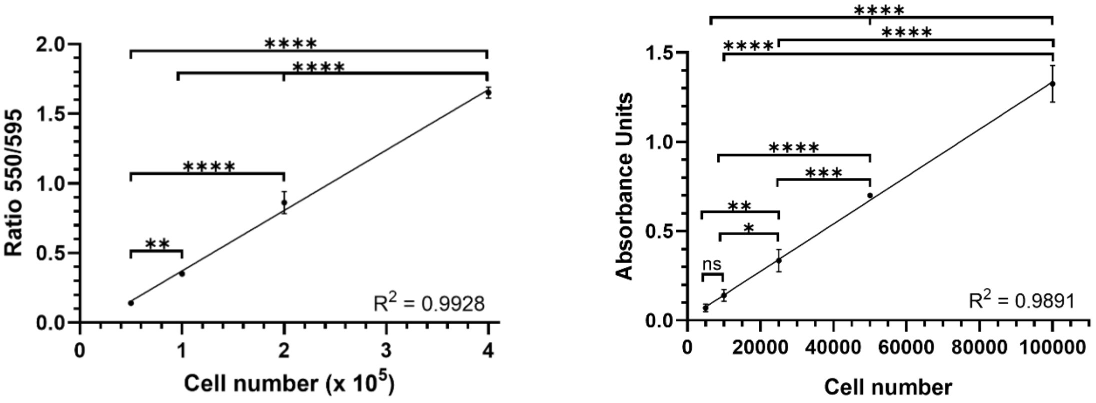

Supplementary Figure 1. (Left) Cell metabolism was assessed using PrestoBlue reagent with graph presented as blank corrected values. Cells seeded in a 2D layer on tissue culture plastic and incubated with PrestoBlue for 1 hour before conducting an absorbance reading of the supernatant. (Right) Cell necrosis was assess using a LDH assay. Cells seeded in a 2D layer on tissue culture plastic then killed using a lysis buffer before the media was retrieved and analysed with an LDH assay. Absorbance values were normalized to the volume of media used in the culture of 3D scaffolds.

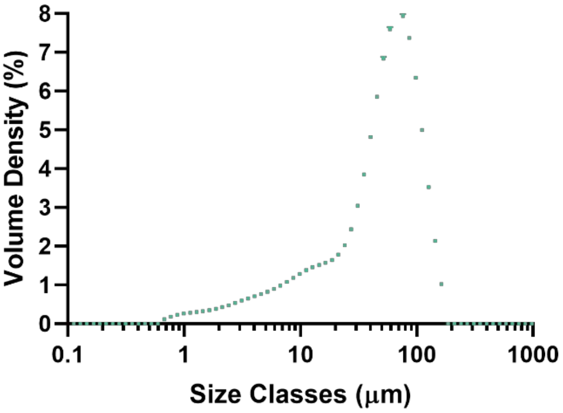

Supplementary Figure 2. Particle size analysis of the large coral particles. Each data point represents the average and standard deviation of n = 10 measurements made up from two technical replicates with five measurements each. Data points without visible error bars means the error was too small to show.

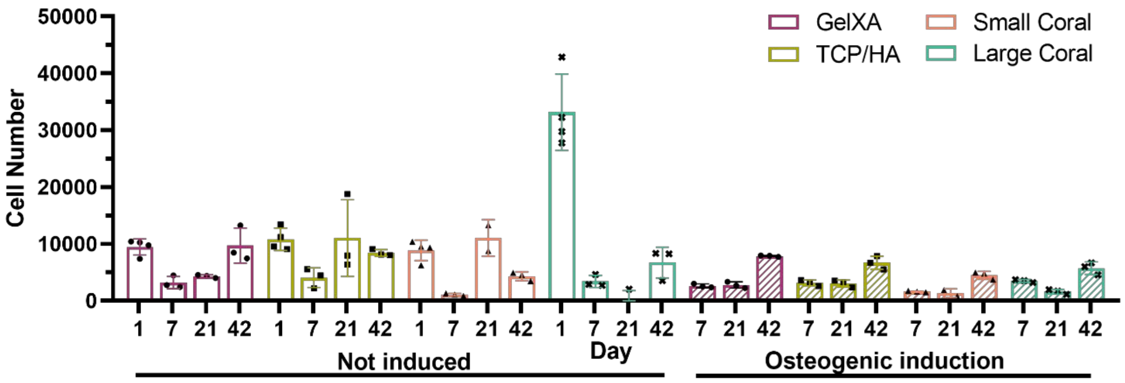

Supplementary Figure 3. LDH levels were tracked across the 6 weeks as a high-throughput and non-destructive to monitor the scaffolds. Scaffolds were printed at 10 million cells per ml therefore each scaffold contained ≈250,000 cells. In the 24 hours post printing all conditions had less than 5% cell death excluding the large coral size which experienced approximately 13% cell death. This was likely due to challenges in printing which required the large coral ink to change from printing with a 23 to 22G needle due to blockages. All remaining time points and conditions display equal or lower cell death which again represents less than 5% cell number from original seeding density.

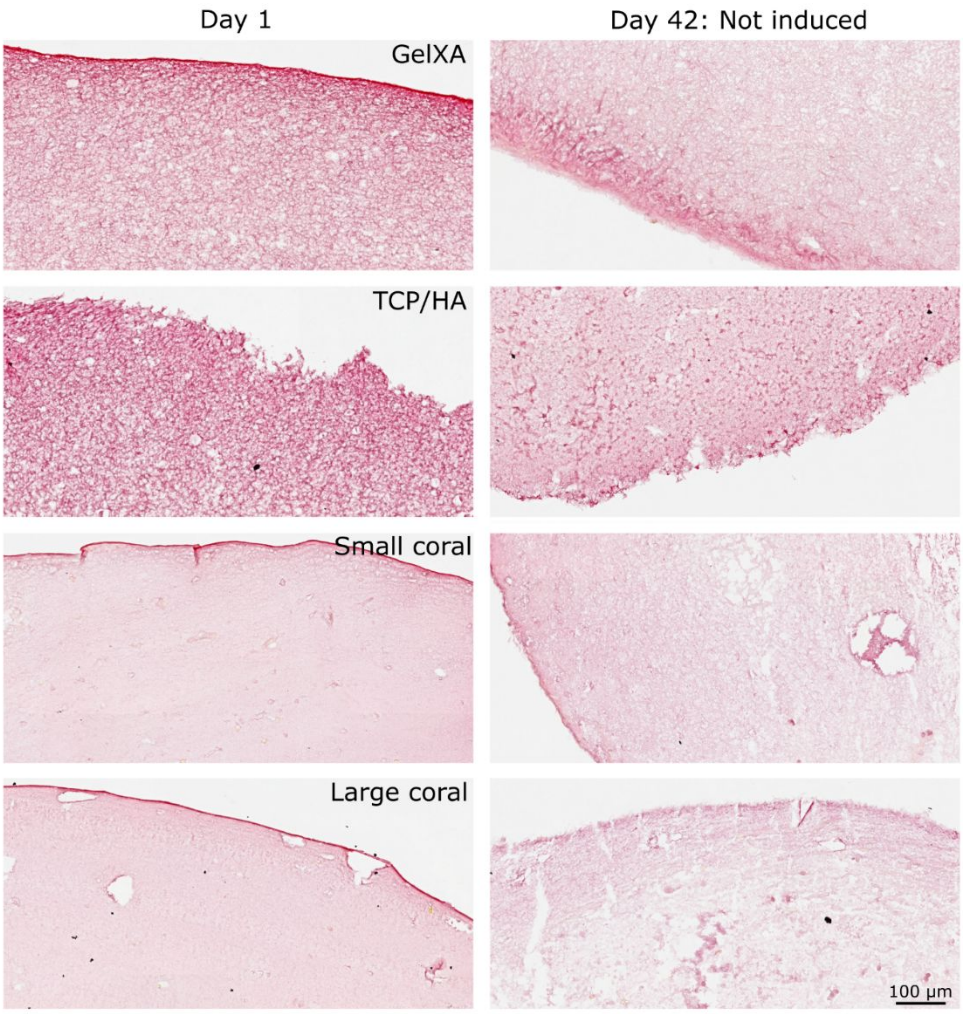

Supplementary Figure 4. Picro Sirius Red staining imaged under light microscopy. Red staining indicates collagen however is also absorbed by the bioinks.

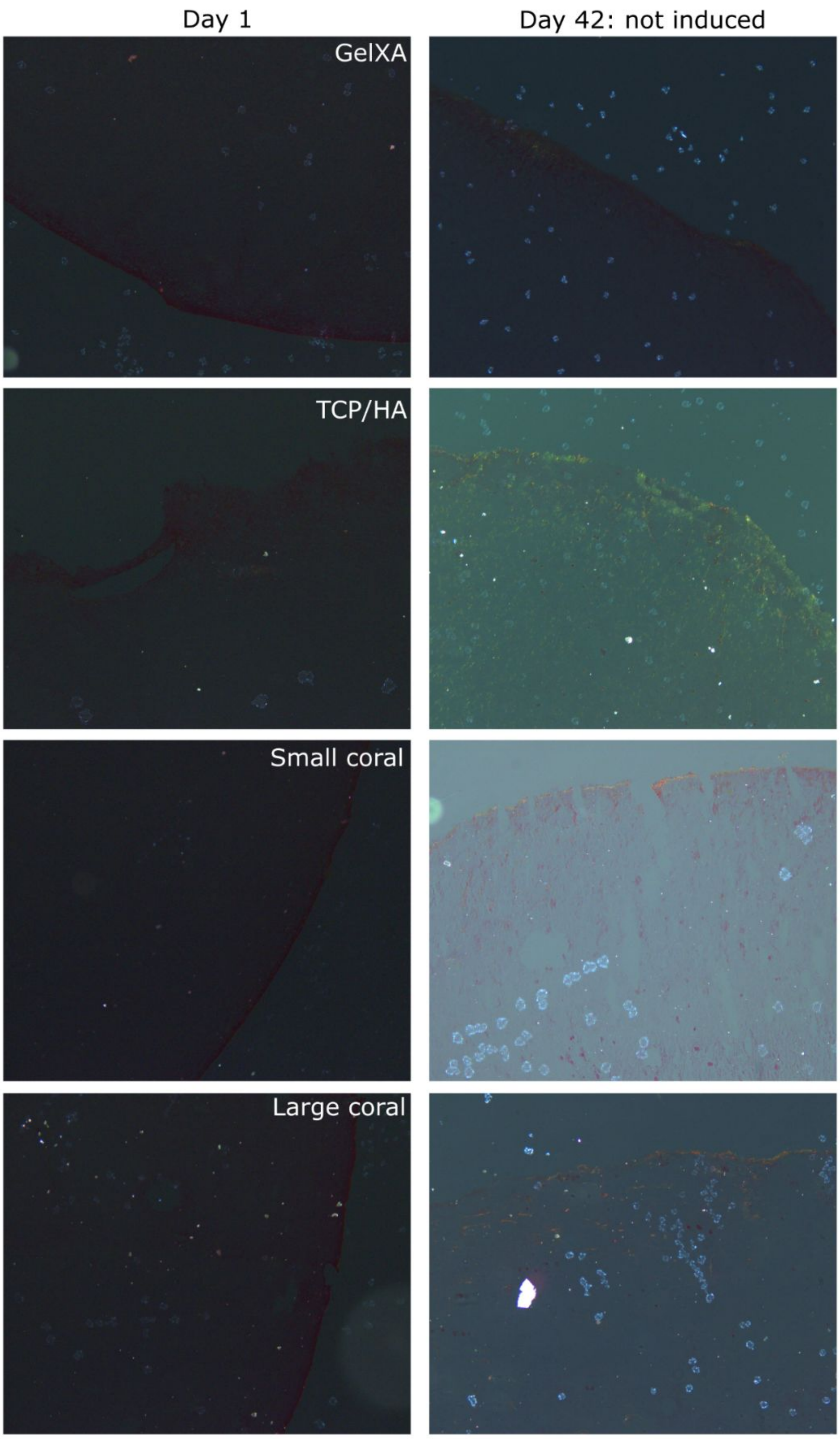

Supplementary Figure 5. Picro Sirius Red staining imaged under polarised light microscopy. Yellow to orange indicate collagen type I.

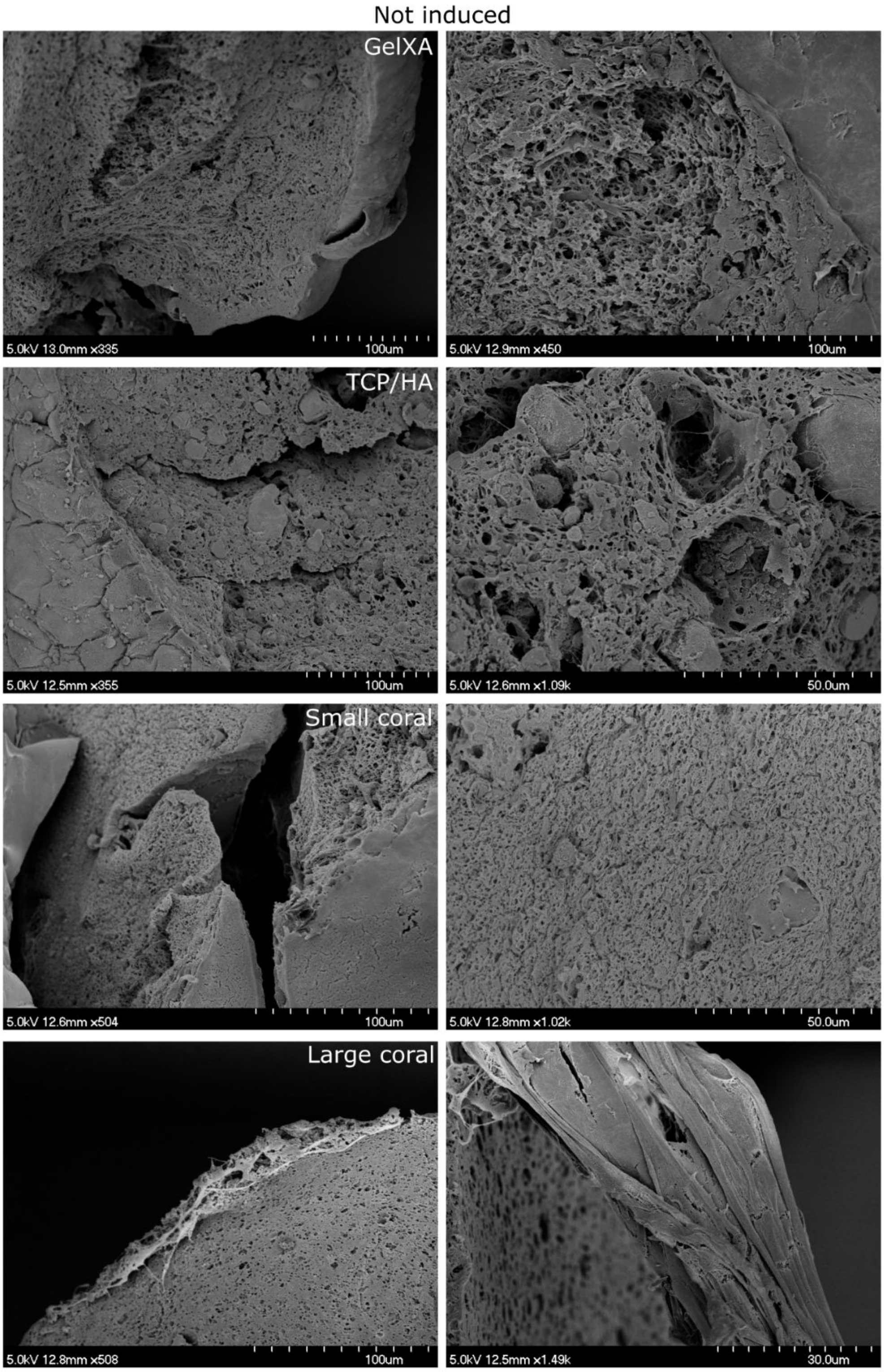

Supplementary Figure 6. SEM imaging of scaffolds after culture for 42 days in non-induction media.

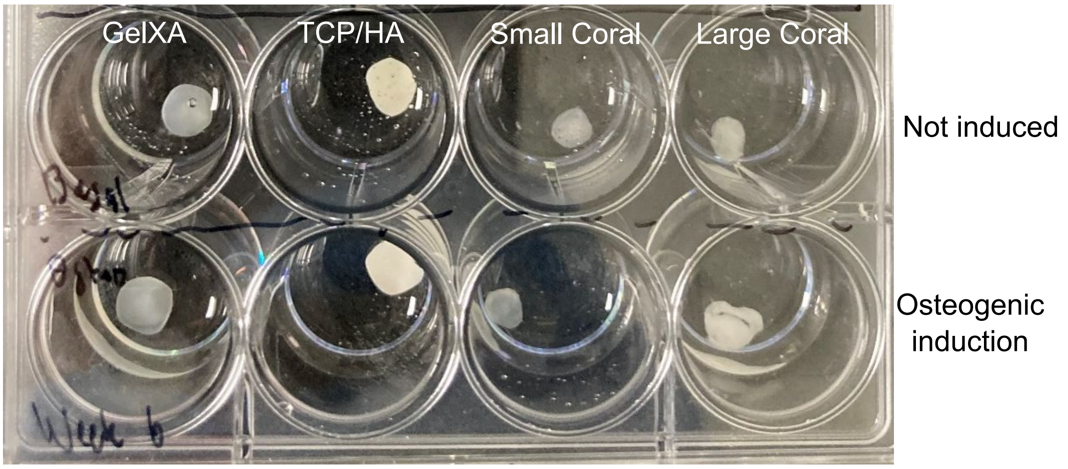

Supplementary Figure 7. Scaffolds fixed then stored in sodium cacodylate buffer for up to 3 months. Opacity of the coral, especially small size coral, was reduced with greater transparency the longer stored in the buffer.

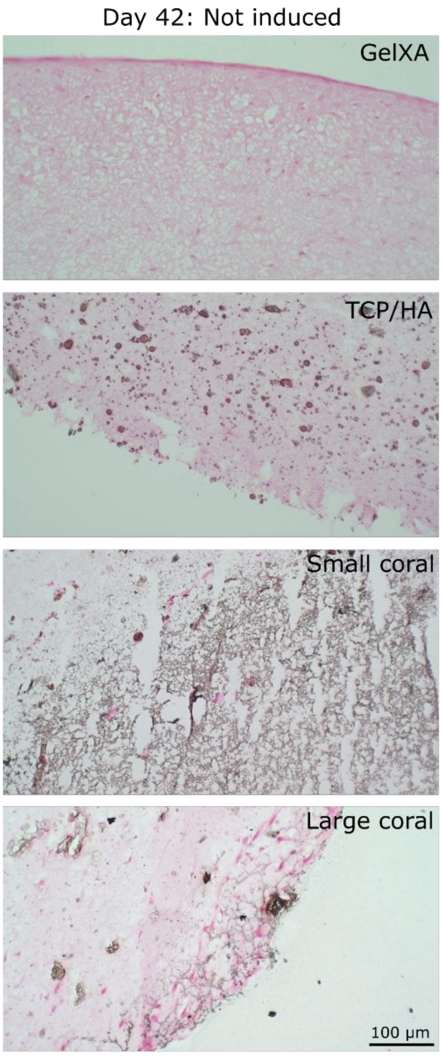

Supplementary Figure 8. Von Kossa staining where brown represents calcium and pink is cytoplasm but also background absorbance by the bioink.

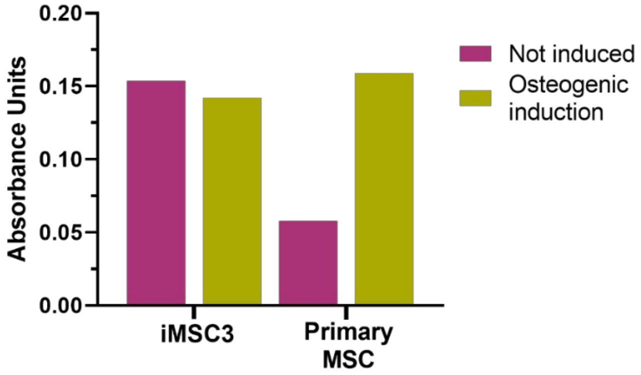

Supplementary Figure 9. ALP content from conditioned media combined with p-nitrophenyl phosphate and read at 405 nm.
